# Association between sleep duration and healthy aging among older adults: evidence from the Behavioral Risk Factor Surveillance System

**DOI:** 10.1186/s12877-026-07181-8

**Published:** 2026-02-21

**Authors:** Liuhong Tian, Wenjing Chen, Shulei Chen, Xiaodan kuang, Jiaming Fang, Mengjia Jin, Hongying Shi

**Affiliations:** 1 Department of Epidemiology and Health Statistics, School of Public Health, Wenzhou Medical University, Wenzhou, Zhejiang Province 325035 China; 2 National Clinical Research Center for Ocular Diseases, Eye Hospitalof Wenzhou Medical University, 618 East Fengqi Rd, Hangzhou, 310020 China

**Keywords:** Sleep duration, Healthy aging, Older adults, BRFSS

## Abstract

**Objective:**

This study aims to explore the association between sleep duration and healthy aging in the older U.S. adults, utilizing a nationally representative sample.

**Methods:**

Participants aged 65 and older from the 2016 Behavioral Risk Factor Surveillance System were eligible for this study. Healthy aging was defined as the absence of major chronic diseases, no physical functional limitations, and good subjective cognitive function and mental health. Logistic regression and restricted cubic spline curve analysis were employed to examine the potential association between sleep duration and healthy aging. Stratified analyses were conducted to examine the interactive effects of sleep duration with smoking, employment status, and other variables on healthy aging.

**Results:**

35,056 older adults (mean age 73.06 ± 5.31 years, 60.5% females) were included, among whom 5,782 (16.5%) achieved healthy aging. Sleep duration exhibited an inverted U-shaped relationship with healthy aging. Compared to those who slept for 7 h, the adjusted ORs (95% CI) of healthy aging for those who slept for ≤ 5 h, 6 h, 8 h, and ≥ 9 h were 0.51 (0.43, 0.59), 0.79 (0.72, 0.87), 0.96 (0.90, 1.03), and 0.65 (0.58, 0.73), respectively. Additionally, there was an interaction between sleep duration and employment & smoking status on healthy aging (*P* interaction = 0.003 & 0.009). Among the unemployed, the adjusted OR (95% CI) for healthy aging among those who slept ≤ 5 h was 0.41 (0.34, 0.49) [*VS* the employed: 0.75 (0.56, 1.01)]; among current and former smokers, the adjusted OR (95% CI) for healthy aging among those who slept ≤ 5 h was 0.37 (0.17, 0.82) and 0.39 (0.29, 0.51) [*VS* never smokers: 0.60 (0.49, 0.73)].

**Conclusion:**

Both short and prolonged sleep duration may adversely impact healthy aging, especially among the unemployed and smokers. Health professionals should advise older adults that 7–8 h of sleep per day may promote overall health. Future research should explore the longitudinal relationship between sleep duration and healthy aging.

**Supplementary Information:**

The online version contains supplementary material available at 10.1186/s12877-026-07181-8.

## Introduction

The number of individuals aged 65 and older in the United States (U.S.) is projected to increase from 58 million in 2022 to 82 million by 2050, a 47% rise; additionally, proportion of the older adults is expected to grow from 17% to 23% [1, 2]. However, living long does not necessarily mean living healthily, as aging often brings chronic diseases, disability, dementia, and mental illness, imposing economic and social burdens on individuals, families, and society [3]. Achieving healthy aging—avoiding chronic diseases and physical limitations, and maintaining good cognitive and mental health—has become a critical concern for governments, researchers, and the public. The United Nations General Assembly’s (UNGA) declaration of 2021–2030 as the Decade of Healthy Aging underscores the global focus on improving the health of older adults [4, 5]. The National Academy of Medicine (NAM) is also launching a grand challenge to promote healthy aging for all [6].

Sleep is essential for human health, and growing evidence links sleep duration to individual health outcomes. Both insufficient and excessive sleep are associated with adverse health outcomes [7, 8], including increased mortality [9, 10], chronic diseases [11, 12], mental illness [13, 14], and cognitive decline [15, 16]. However, in modern society, many older adults have sleep problems. A study involving 11 million participants with objective sleep records found that 29.2% of the older adults slept less than 7 h, while 6.8% slept more than 9 h [17]. In the U.S., 26.0% of the older adults slept less than 7 h [18]. The relationship between sleep duration and overall health requires further exploration. A recent study among American nurses suggested that those sleeping 7 h had the highest odds of healthy aging [19]. However, this study focused on a specific occupational group and did not account for different genders and occupations. Analyzing the relationship between sleep duration and overall health among a more representative population can provide better evidence for recommending optimal sleep duration.

Therefore, this study, utilizing the 2016 Behavioral Risk Factor Surveillance System (BRFSS) database, aims to examine the association between sleep duration and healthy aging among older adults in a nationally representative sample of the U.S. population, and to explore the heterogeneity of this association across different population characteristics, with the ultimate goal of providing a scientific basis for improving the sleep duration of older adults.

## Methods

### Study design

The BRFSS, initiated by the Centers for Disease Control and Prevention (CDC) in 1984, is a large-scale cross-sectional telephone survey (landline and cell phone) encompassing all 50 states and the District of Columbia (DC). Employing multi-stage sampling, it targets adults aged ≥ 18 years. The survey collects data on behavioral factors associated with disease development and mortality, including smoking status, alcohol consumption, sleep duration, physical activity, dietary habits, and chronic diseases. The median response rate among the 50 states and the DC for the 2016 survey was 47.0%, ranging from 29.0% in Alabama to 58.0% in Colorado. Data have been shown to align with other national surveys based on self-reported behaviors [20, 21]. Approved by the CDC Institutional Review Board, the survey is publicly accessible. This study adhered to the Strengthening the Reporting of Observational Studies in Epidemiology (STROBE) guidelines.

This secondary analysis used data from the BRFSS standard core module for 2016. Among 477,665 surveyed adults, 171,833 aged 65 and above were potential subjects. After excluding those with missing sleep duration (*n* = 11,563) and healthy aging information (*n* = 126,520), the final sample size was 35,056.

### Assessment of sleep duration

Sleep duration was self-reported by asking, “On average, how many hours of sleep do you get in a 24-h period (where sleep duration is an integer)?” This measure, consistent with the Pittsburgh Sleep Quality Index (PSQI) [22], is widely used in BRFSS research [15, 18]. In addition, the self-reported sleep duration strongly correlated with sleep duration recorded in sleep diaries (*r*_s_=0.79, *P* < 0.001) [23]. Based on previous studies [19], sleep duration was categorized into ≤ 5 h, 6 h, 7 h, 8 h, and ≥ 9 h.

### Assessment of healthy aging

Currently, there is no standard definition of healthy aging [24]. Based on previous studies on the relationship between sleep duration, night shifts, and healthy aging [19, 25, 26], healthy aging was defined as the absence of major chronic diseases, no physical function limitations, and good subjective cognitive function and mental health. Otherwise, it was categorized as normal aging.

#### Absence of major chronic disease

Major chronic diseases were identified through self-reports by participants regarding diagnoses of heart attack, angina or coronary heart disease, stroke, diabetes, cancer other than skin, chronic obstructive pulmonary disease (including emphysema and chronic bronchitis), arthritis (including rheumatoid arthritis, gout, lupus, and fibromyalgia), kidney disease, and asthma. Those without any of these conditions were considered free of major chronic diseases. This method is widely used in BRFSS research [11, 27].

#### Absence of physical function limitation

Physical function limitations were assessed through questions on five types of disabilities: hearing disability, vision disability, mobility disability, self-care disability, and independent living disability. Respondents were asked if they had serious difficulty hearing, seeing even when wearing glasses, walking or climbing stairs, dressing or bathing, or doing errands alone such as visiting a doctor’s office or shopping due to physical, mental, or emotional condition. Those who answered “no” to any question were considered to have no physical function limitation [28, 29]. This measure aligns with the International Classification of Functioning, Disability and Health (ICF) [30] and has been incorporated into the American Community Survey (ACS) [31].

#### Good subjective cognitive function

Before asking about subjective cognitive function, participants were told that “The next few questions ask about difficulties in thinking or remembering that can make a big difference in everyday activities. This does not refer to occasionally forgetting your keys or the name of someone you recently met, which is normal. This refers to confusion or memory loss that is happening more often or getting worse, such as forgetting how to do things you’ve always done or forgetting things that you would normally know. We want to know how these difficulties impact you”. Then, the participants were asked “During the past 12 months, have you experienced confusion or memory loss that is happening more often or is getting worse?” The respondents who answered no were designated as having good subjective cognitive function [32]. The questions were developed using a multi-step process which included a scientific literature review [33]. Evidence also suggests that even occasional or single instances of self-reported memory complaints can predict future cognitive impairment and dementia [34].

#### Good mental health

Mental health was assessed with two questions. Participants were asked, “how many days during the past 30 days was your mental health not good?” [13]. Reporting 14 or more days of poor mental health was considered impaired mental health, based on guidelines for frequent mental distress research [32]. This threshold is consistent with the American Psychiatric Association’s (APA) guidelines for the required symptom duration for a depression diagnosis [35] and is commonly used by physicians and researchers to identify clinical depression [36]. Additionally, participants were asked, “Have you ever been told that you have depressive disorder (including depression, major depression, dysthymia, or minor depression)?”. Those who answered “no” to both questions were considered to have good mental health [13, 37]. Prior research utilizing BRFSS data has employed a similar definition to identify the presence of depression [13, 37, 38].

### Assessment of covariates

Based on prior BRFSS studies on sleep duration [11, 28, 32, 39, 40], we included the following potential covariates: age group (65–69, 70–74, ≥ 75 years), sex (men, women), race/ethnicity (White, Black, Hispanic, American Indian/Alaska Native (AI/AN), Asian, Other race/Multiracial), marital status (married/living with partner, previously married, never married), educational attainment (< high school graduate, high school graduate/General Educational Development (GED), some college/technical school, college graduate), employment status (employed, unemployed, unable to work, student/homemaker/retired), smoking status (current smoker, former smoker, never smoker), leisure-time physical activity (yes, no), and Body Mass Index (BMI). Based on the World Health Organization (WHO) standards [10], BMI was categorized into four groups: underweight (BMI < 18.5 kg/m²), normal weight (18.5–24.9 kg/m²), overweight (25.0–29.9 kg/m²), and obese (≥ 30.0 kg/m²). Smoking status was obtained by asking participants “Have you smoked at least 100 cigarettes in your entire life? (If you smoke, continue to ask ‘In the past 12 months, have you stopped smoking for one day or longer because you were trying to quit smoking?’). Smoking status was categorized into three groups: current smoker, former smoker, and never smoker. Employment status was assessed by asking, “Are you currently…? (If more than one, select the category which best describes you)”. Based on previous research [28, 40], we combined “employed for wages and “self-employed” into “the employed,” merged “out of work for 1 year or more” and “out of work for less than 1 year” into “the unemployed,” grouped “homemaker” and “retired” into a single category, and classified the remainder as “unable to work”. Leisure-time physical activity was assessed by asking, “During the past month, other than your regular job, did you participate in any physical activities or exercises such as running, calisthenics, golf, gardening, or walking for exercise?” Responses were categorized as yes or no [28].

### Statistical analysis

Quantitative data were expressed as mean ± standard deviation or median (interquartile range), and count data as frequency and percentage. The Chi-square test was used to compare nominal data, and the Kruskal-Wallis test was used to compare ordinal data. Multivariate logistic regression models and restricted cubic spline curve analysis were used to explore the relationship between daily sleep duration and healthy aging, with results expressed as odds ratios (ORs) and 95% confidence intervals (CI). Model 1 adjusted for age, sex, race/ethnicity, marital status, educational attainment, and employment status; Model 2 further adjusted for smoking status, leisure-time physical activity, and BMI.

To examine the consistency of the association between sleep duration and healthy aging across different populations, we conducted stratified analyses. For Race/ethnicity, due to small sample sizes of Hispanic, AI/AN, Asian, and Other race/Multiracial, we combined them into Hispanic and Other race. In employment status, the small sample sizes of the unable to work and homemaker/retired groups led to their combination with the unemployed group. We calculated the *P* value for the multiplicative interaction effect using the likelihood ratio test. To verify the robustness of our results, we perform several sensitivity analyses. First, given that 18.51% of the data for annual household income were missing, we further adjusted for this variable in a separate model to assess the stability of the association between sleep duration and healthy aging. Second, to address the impact of missing values (Supplementary Table 1 shows the number and proportion of missing covariates), we conducted 10 multiple imputations based on chained equations for missing covariates using the R package “mice” [41]. Third, we calculated the *E* value for the statistically significant effect size of sleep duration on healthy aging. It represents the minimum strength of association, on the risk ratio scale, that an unmeasured confounder would need to have with both the treatment and outcome, conditional on the measured covariates, to explain away the treatment-outcome association [42].

All analyses were performed using Empower^®^ 4.0 and R 4.2.2. A two-sided *P* < 0.05 was considered statistically significant.

## Results

### Basic characteristics by sleep duration

The study included 35,056 older adults with an average age of 73.06 ± 5.31 years (60.5% females). Among them, 2701 (8.3%) slept ≤ 5 h, 6026 (14.6%) slept 6 h, 9958 (19.6%) slept 7 h, 12,118 (18.6%) slept 8 h, and 4253 (11.3%) slept ≥ 9 h. Table 1 presents the baseline characteristics of the subjects by sleep duration. Compared to those who slept 7 h, individuals with shorter or longer sleep duration were more likely to be divorced/widowed, current smokers, physically inactive, and either underweight or obese. Participants who slept 7 h were more likely to be employed (all *P* < 0.05).

**Table 1:** Basic characteristics of the study population by sleep duration

Characteristics	Sleep duration, h/d					*P*-value
	≤ 5	6	7	8	≥ 9	
Age group, y, *n* (%)						0.671
65–69	873 (32.3)	1973 (32.7)	3314 (33.3)	4084 (33.7)	1395 (32.8)	
70–74	700 (25.9)	1559 (25.9)	2467 (24.8)	3007 (24.8)	1064 (25.0)	
≥ 75	1128 (41.8)	2494 (41.4)	4177 (41.9)	5027 (41.5)	1794 (42.2)	
Sex, *n* (%)						0.836
Male	1067 (39.5)	2387 (39.6)	3885 (39.0)	4798 (39.6)	1700 (40.0)	
Female	1634 (60.5)	3639 (60.4)	6073 (61.0)	7319 (60.4)	2553 (60.0)	
Race/ethnicity, *n* (%)						0.563
White	2268 (86.0)	5136 (87.7)	8501 (87.8)	10,326 (87.7)	3641 (88.6)	
Black	176 (6.7)	344 (5.9)	558 (5.8)	693 (5.9)	210 (5.1)	
Hispanic	114 (4.3)	232 (4.0)	378 (3.9)	459 (3.9)	162 (3.9)	
AI/AN	38 (1.4)	57 (1.0)	106 (1.1)	119 (1.0)	46 (1.1)	
Asian	30 (1.1)	53 (0.9)	87 (0.9)	114 (1.0)	34 (0.8)	
Other race/Multiracial	12 (0.5)	37 (0.6)	50 (0.5)	59 (0.5)	17 (0.4)	
Marital status, *n* (%)						< 0.001
Married/living with partner	1027 (38.3)	2824 (47.1)	5348 (53.9)	6403 (53.1)	1995 (47.2)	
Previously married	1474 (54.9)	2838 (47.3)	4051 (40.8)	5030 (41.7)	2027 (48.0)	
Never married	183 (6.8)	338 (5.6)	527 (5.3)	635 (5.3)	203 (4.8)	
Educational attainment, *n* (%)						0.561
< High school graduate	225 (8.4)	484 (8.1)	761 (7.7)	954 (7.9)	355 (8.4)	
High school graduate/GED	793 (29.5)	1791 (29.8)	3062 (30.9)	3707 (30.7)	1259 (29.7)	
Some college/technical school	749 (27.9)	1615 (26.9)	2610 (26.3)	3139 (26.0)	1107 (26.1)	
College graduate	920 (34.2)	2118 (35.3)	3481 (35.1)	4268 (35.4)	1516 (35.8)	
Employment status, *n* (%)						< 0.001
Employed	361 (13.4)	1131 (18.8)	1986 (20.0)	1824 (15.1)	397 (9.4)	
Unemployed	42 (1.6)	84 (1.4)	106 (1.1)	125 (1.0)	33 (0.8)	
Unable to work	300 (11.2)	301 (5.0)	228 (2.3)	384 (3.2)	262 (6.2)	
Homemaker/retired	1988 (73.9)	4488 (74.6)	7599 (76.6)	9736 (80.7)	3541 (83.7)	
Smoking status, *n* (%)						< 0.001
Current smoker	177 (6.6)	290 (4.8)	359 (3.6)	452 (3.8)	195 (4.6)	
Former smoker	1231 (45.9)	2705 (45.2)	4334 (43.8)	5557 (46.2)	2143 (50.7)	
Never smoker	1277 (47.6)	2993 (50.0)	5200 (52.6)	6033 (50.1)	1887 (44.7)	
Leisure-time physical activity, *n* (%)						< 0.001
Yes	1536 (57.0)	4066 (67.6)	7637 (76.9)	8781 (72.6)	2559 (60.3)	
No	1160 (43.0)	1952 (32.4)	2300 (23.2)	3316 (27.4)	1685 (39.7)	
BMI (kg/m^2^), *n* (%)						**< 0.001**
< 18.5	56 (2.2)	103 (1.8)	132 (1.4)	191 (1.7)	72 (1.8)	
18.5–24.9	697 (27.4)	1678 (29.6)	3205 (34.0)	3762 (32.6)	1277 (31.4)	
25.0-29.9	878 (34.5)	2147 (37.9)	3732 (39.5)	4590 (39.7)	1547 (38.0)	
≥ 30.0	916 (36.0)	1744 (30.8)	2371 (25.1)	3012 (26.1)	1178 (28.9)	

Categorical data are reported as the number and percentage of participants

*Abbreviations*: *AI/AN* American Indian/Alaska Native, *BMI* Body mass index (calculated as weight in kilograms divided by the square of height in meters)

### Association between sleep duration and healthy aging and its dimensions

**Fig. 1:**
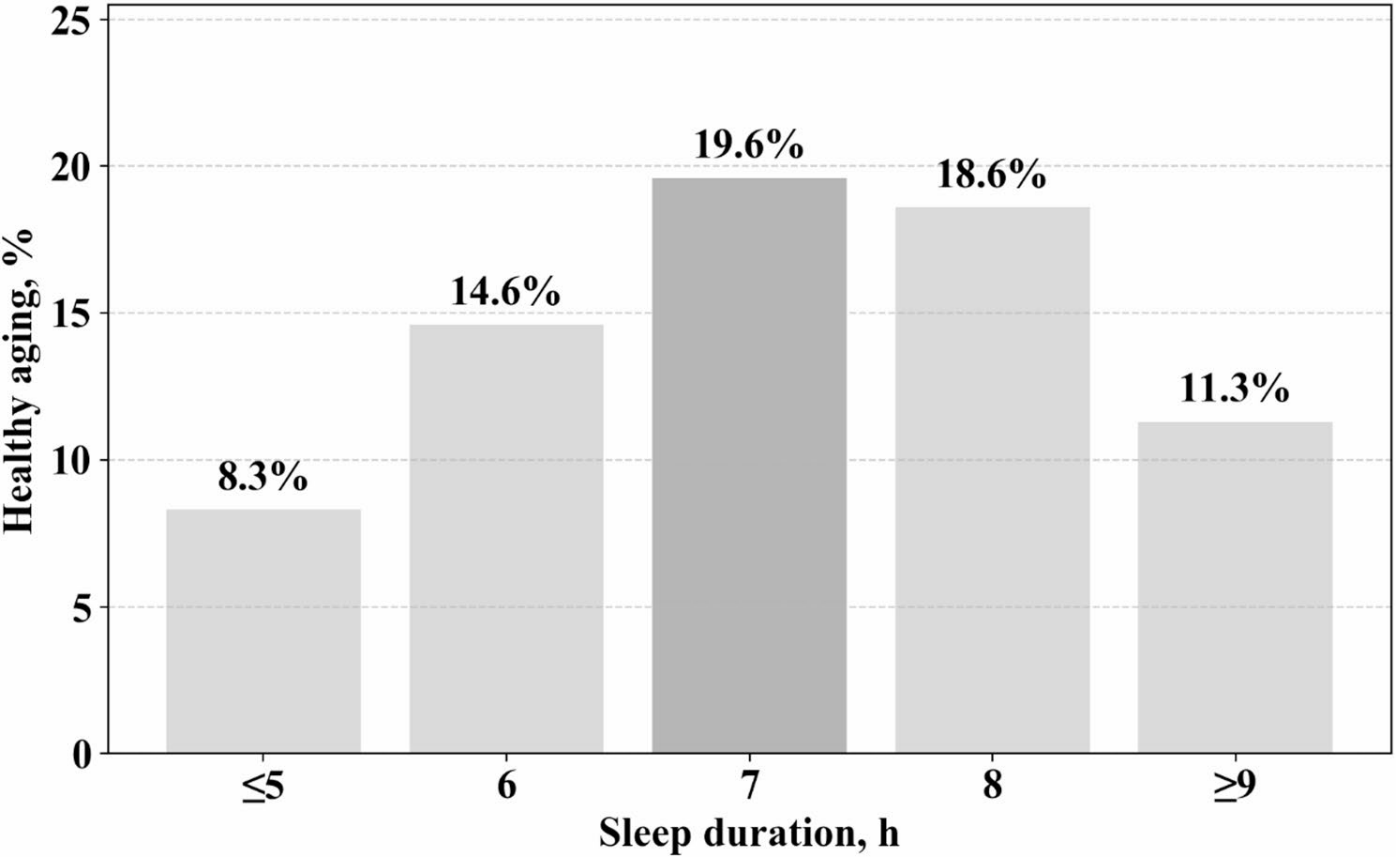
Healthy aging rates according to sleep duration

Figure 1 illustrates the healthy aging rate across different sleep durations. The number of older adults achieving healthy aging was 224 (8.3%) for ≤ 5 h, 878 (14.6%) for 6 h, 1948 (19.6%) for 7 h, 2250 (18.6%) for 8 h, and 482 (11.3%) for ≥ 9 h, respectively. Table 2 presents the association between sleep duration and healthy aging. In the unadjusted model, the ORs (95% CI) for ≤ 5 h, 6 h, 8 h, and ≥ 9 h compared to 7 h were 0.37 (0.32, 0.43), 0.70 (0.64, 0.77), 0.94 (0.88, 1.00), and 0.53 (0.47, 0.58), respectively. After adjusting for all confounding factors (Model 2), the adjusted ORs (95% CI) remained statistically significant.

**Table 2:** Association of sleep duration with healthy aging

Sleep duration, h/d	No.	Healthy aging, No. (%)	OR(95%CI), *P*		
			Crude model, (*n* = 35,056)	Adjusted model I (*n* = 33,909)	Adjusted model Ⅱ (*n* = 30,648)
≤ 5	2,701	224 (8.3)	**0.37 (0.32**,**0.43)**,** < 0.001**	**0.44 (0.38**,** 0.51)**,** < 0.001**	**0.51 (0.43**,** 0.59)**,** < 0.001**
6	6,026	878 (14.6)	**0.70 (0.64**,**0.77)**,** < 0.001**	**0.74 (0.68**,** 0.81)**,** < 0.001**	**0.79 (0.72**,** 0.87)**,** < 0.001**
7	9,958	1948 (19.6)	1.00	1.00	1.00
8	12,118	2250 (18.6)	0.94 (0.88,1.00), 0.060	0.99 (0.92, 1.06), 0.644	0.96 (0.90, 1.03),0.480
≥ 9	4,253	482 (11.3)	**0.53 (0.47**,**0.58)**,** < 0.001**	**0.60 (0.53**,** 0.67)**,** < 0.001**	**0.65 (0.58**,** 0.73)**,** < 0.001**

*Abbreviations*: *AI/AN* American Indian/Alaska Native, *BMI* Body mass index (calculated as weight in kilograms divided by the square of height in meters), *OR* Odds ratio, *CI* Confidence interval

Model I was adjusted for age group (65–69, 70–74, ≥ 75 years), sex (male, female), race/ethnicity (White, Black, Hispanic, American Indian/Alaska Native (AI/AN), Asian, Other race/Multiracial), marital status (married/living with partner, previously married, never married), educational attainment (< high school graduate, high school graduate/GED, some college/technical school, college graduate), and employment status (employed, unemployed, unable to work, student/homemaker/retired)

Model II was additionally adjusted for smoking status (current smoker, former smoker, never smoker), leisure-time physical activity (yes, no), and BMI (< 18.5 kg/m^2^, 18.5–24.9 kg/m^2^, 25.0–25.9 kg/m^2^), ≥30.0 kg/m^2^)

Supplemental Table 2 shows the relationship between sleep duration and the four dimensions of healthy aging. In the fully adjusted model, the adjusted ORs (95% CI) for “no major chronic disease” were 0.59 (0.52, 0.68) for ≤ 5 h, 0.83 (0.76, 0.90) for 6 h, 1.06 (0.99, 1.13) for 8 h, and 0.76 (0.69, 0.84) for ≥ 9 h. For “no limitation of physical function”, the ORs (95% CI) were 0.42 (0.38, 0.46), 0.73 (0.68, 0.79), 0.87 (0.82, 0.92), and 0.53 (0.49, 0.58), respectively. For “good subjective cognitive function”, the ORs (95% CI) were 0.56 (0.49, 0.65), 0.87 (0.78, 0.98), 0.99 (0.89, 1.09), and 0.60 (0.53, 0.68), respectively. For “good mental health”, the ORs (95% CI) were 0.49 (0.44, 0.55), 0.82 (0.74, 0.89), 0.98 (0.90, 1.06), and 0.56 (0.51, 0.62), respectively.

**Fig. 2:**
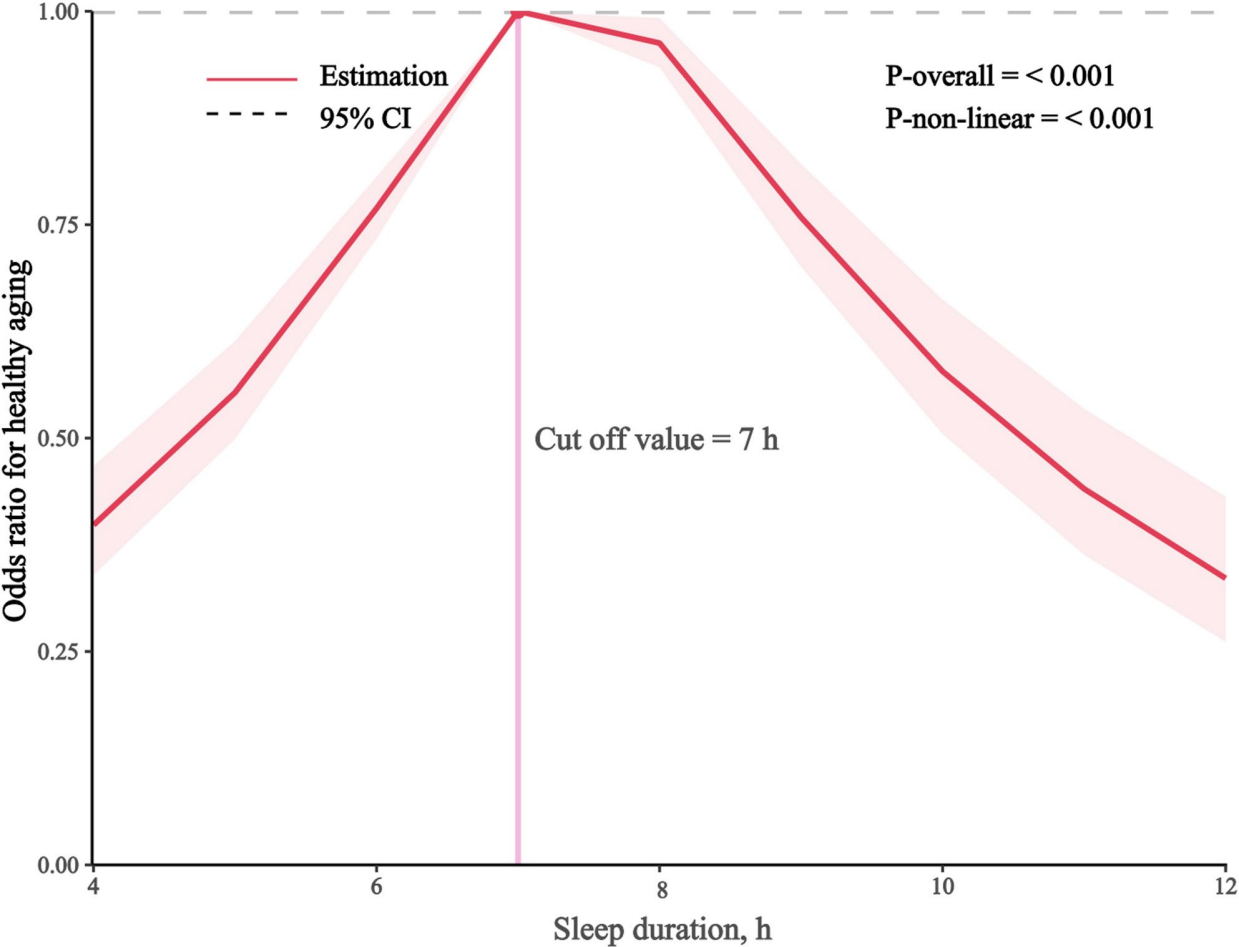
Spline curve for the association of sleep duration with healthy aging

Restricted cubic spline curve analysis revealed an inverted U-shaped relationship between sleep duration and healthy aging (Fig. 2 and Supplementary Fig. 1). Similar associations were observed for “no major chronic disease”, “no limitation of physiological function”, “good subjective cognitive function”, and “good mental health” (all *P* nonlinearity < 0.001).

Adjusted for age group (65–69, 70–74, ≥ 75 years), sex (male, female), race/ethnicity (White, Black, Hispanic, American Indian/Alaska Native (AI/AN), Asian, Other race/Multiracial), marital status (married/living with partner, previously married, never married), education (< high school graduate, high school graduate/GED, some college/technical school, college graduate), employment status (employed, unemployed, unable to work, homemaker/retired), smoking status (current smoker, former smoker, never smoker), leisure-time physical activity (yes, no), BMI (< 18.5 kg/m^2^, 18.5–24.9 kg/m^2^, 25.0–25.9 kg/m^2^, ≥ 30.0 kg/m^2^).

### Stratified analysis

Table 3 presents the stratified analysis by age, sex, race/ethnicity, marital status, employment status, smoking status, leisure-time physical activity, and BMI. Across different subgroups of individuals, the association between sleep duration and healthy aging remains largely consistent. However, there was a significant interaction between sleep duration and employment status on healthy aging (*P* interaction = 0.003). Among the unemployed, those who slept ≤ 5 h had the lowest odds of achieving healthy aging compared to those who slept 7 h [adjusted OR (95% CI) = 0.41 (0.34, 0.49)]. Among the employed, those who slept ≥ 9 h had the lowest odds (adjusted OR (95% CI) = 0.75 (0.57, 0.98)). There was also an interaction between sleep duration and smoking status (*P* interaction = 0.009). Among current and former smokers, those who slept ≤ 5 h had the lowest odds of achieving healthy aging compared to those who slept 7 h [current smokers: adjusted OR (95% CI) = 0.37 (0.17, 0.82); former smokers: adjusted OR (95% CI) = 0.39 (0.29, 0.51)]. Among non-smokers, the adjusted OR (95% CI) for those who slept ≤ 5 h was 0.60 (0.49, 0.73).

**Table 3:** Association of healthy aging with sleep duration, stratification analyses

Subgroup	*n*	OR(95%CI), *P*					*P* for interaction
		Sleep duration, h/day					
		≤ 5	6	7	8	≥ 9	
Age group, y							**0.032**
65–69	11,639	0.46 (0.35, 0.61), < 0.001	0.72 (0.61, 0.86), < 0.001	1.00	0.92 (0.81, 1.05), 0.231	0.75 (0.62, 0.91), 0.004	
70–74	8,797	0.46 (0.33, 0.64), < 0.001	0.90 (0.75, 1.08), 0.256	1.00	1.20 (1.03, 1.39), 0.017	0.60 (0.47, 0.77), < 0.001	
≥ 75	14,620	0.55 (0.44, 0.69), < 0.001	0.78 (0.68, 0.91), 0.001	1.00	0.99 (0.89, 1.11), 0.867	0.61 (0.51, 0.73), < 0.001	
Sex							0.916
Male	13,837	0.48 (0.37, 0.61), < 0.001	0.82 (0.71, 0.95), 0.009	1.00	1.01 (0.90, 1.13), 0.885	0.63 (0.52, 0.76), < 0.001	
Female	21,218	0.52 (0.43, 0.64), < 0.001	0.78 (0.69, 0.88), < 0.001	1.00	1.02 (0.93, 1.12), 0.743	0.67 (0.58, 0.78), < 0.001	
Race/ethnicity ^*a*^							0.346
White	29,872	0.48 (0.41, 0.57), < 0.001	0.80 (0.73, 0.89), < 0.001	1.00	1.02 (0.94, 1.10), 0.645	0.67 (0.59, 0.76), < 0.001	
Black	1,981	0.32 (0.15, 0.66), 0.002	0.67 (0.44, 1.01), 0.058	1.00	0.78 (0.57, 1.08), 0.140	0.39 (0.21, 0.72), 0.002	
Hispanic and Other race	2,204	1.08 (0.61, 1.91), 0.788	0.78 (0.50, 1.20), 0.256	1.00	1.20 (0.87, 1.67), 0.271	0.58 (0.32, 1.03), < 0.062	
Marital status							
Married/living with partner	17,597	0.49 (0.39, 0.62), < 0.001	0.87 (0.76, 0.98), 0.027	1.00	1.03 (0.93, 1.13), 0.581	0.67 (0.57, 0.78), < 0.001	0.606
Previously married	15,420	0.49 (0.39, 0.63), < 0.001	0.72 (0.62, 0.84), < 0.001	1.00	0.98 (0.87, 1.11), 0.774	0.65 (0.54, 0.78), < 0.001	
Never married	1,886	0.58 (0.33, 1.02), 0.057	0.67 (0.45, 0.99), 0.044	1.00	1.10 (0.81, 1.49), 0.533	0.54 (0.32, 0.91), 0.026	
Educational attainment							0.549
< High school graduate	2,779	0.76 (0.44, 1.31), 0.319	0.77 (0.52, 1.13), 0.178	1.00	1.12 (0.83, 1.50), 0.481	0.44 (0.25, 0.74), 0.002	
High school graduate/GED	10,612	0.46 (0.34, 0.62), < 0.001	0.82 (0.70, 0.98), 0.028	1.00	0.97 (0.85, 1.11), 0.672	0.70 (0.57, 0.86), < 0.001	
Some college/technical school	9,220	0.58 (0.44, 0.76), < 0.001	0.76 (0.63, 0.92), 0.004	1.00	1.05 (0.91, 1.21), 0.498	0.65 (0.52, 0.81), < 0.001	
College graduate	12,303	0.43 (0.33, 0.57), < 0.001	0.81 (0.69, 0.94), 0.007	1.00	1.02 (0.90, 1.15), 0.803	0.67 (0.55, 0.81), < 0.001	
Employment status ^*b*^							**0.003**
Employed	5,699	0.75 (0.56, 1.01), 0.059	0.98 (0.82, 1.17), 0.855	1.00	1.00 (0.86, 1.16), 0.976	0.75 (0.57, 0.98), 0.038	
Unemployed	29,217	0.41 (0.34, 0.49), < 0.001	0.72 (0.64, 0.80), < 0.001	1.00	1.00 (0.92, 1.09), 0.940	0.62 (0.54, 0.70), < 0.001	
Smoking status							**0.009**
Current smoker	1,473	0.37 (0.17, 0.82), 0.015	0.35 (0.18, 0.68), 0.002	1.00	1.11 (0.73, 1.69), 0.630	0.77 (0.42, 1.41), 0.403	
Former smoker	15,970	0.39 (0.29, 0.51), < 0.001	0.72 (0.62, 0.83), < 0.001	1.00	0.96 (0.86, 1.08), 0.516	0.63 (0.53, 0.76), < 0.001	
Never smoker	17,390	0.60 (0.49, 0.73), < 0.001	0.88 (0.78, 1.00), 0.044	1.00	1.05 (0.95, 1.15), 0.352	0.66 (0.56, 0.77), < 0.001	
Leisure-time physical activity							0.302
Yes	24,579	0.53 (0.45, 0.64), < 0.001	0.81 (0.73, 0.90), < 0.001	1.00	1.02 (0.94, 1.11), 0.579	0.69 (0.60, 0.79), < 0.001	
No	10,413	0.40 (0.29, 0.55), < 0.001	0.70 (0.56, 0.87), 0.001	1.00	0.95 (0.80, 1.14), 0.581	0.54 (0.42, 0.69), < 0.001	
BMI (kg/m^2^)							0.565
< 25.0	11,173	0.41 (0.32, 0.54), < 0.001	0.78 (0.67, 0.91), 0.001	1.00	1.05 (0.93, 1.17), 0.433	0.62 (0.52, 0.74), < 0.001	
25.0-29.9	12,894	0.58 (0.46, 0.74), < 0.001	0.82 (0.71, 0.95), 0.010	1.00	1.02 (0.91, 1.14), 0.768	0.67 (0.56, 0.81), < 0.001	
≥ 30.0	9,221	0.52 (0.37, 0.72), < 0.001	0.76 (0.61, 0.95), 0.014	1.00	0.93 (0.78, 1.11), 0.415	0.67 (0.51, 0.89), 0.005	

Abbreviations: AI/AN: American Indian/Alaska Native; BMI, body mass index (calculated as weight in kilograms divided by the square of height in meters); OR, odds ratio; CI, confidence interval

Adjusted for age group (65–69, 70–74, ≥ 75 years), sex (men, women), race/ethnicity (White, Black, Hispanic, American Indian/Alaska Native (AI/AN), Asian, Other race/Multiracial), marital status (married/living with partner, previously married, never married), educational attainment (< high school graduate, high school graduate/GED, some college/technical school, college graduate), employment status (employed, unemployed, unable to work, homemaker/retired), smoking status (current smoker, former smoker, never smoker), leisure-time physical activity (yes, no), and BMI (< 18.5 kg/m^2^, 18.5–24.9 kg/m^2^, 25.0–25.9 kg/m^2^), ≥ 30.0 kg/m^2^)

^a^ The AI/AN, Asian, Other race/Multiracial and Hispanic groups were combined due to the small sample size of the AI/AN, Asian, Other race/Multiracial group

^b^ Due to the small sample sizes of the unable to work and homemaker/retired groups, the unable to work and homemaker/retired groups were combined with the unemployed group

### Sensitivity analysis

Several sensitivity analyses were conducted. First, we further adjusted the annual household income based on model 2 and found that the association between sleep duration and healthy aging remained consistent (Supplemental Table 3). Second, after applying multiple imputations for missing data, the “inverted U-shaped association” between sleep duration and healthy aging persisted (Supplemental Table 4). Finally, the *E* value for ≤ 5 h of sleep and healthy aging was 2.78 (upper confidence interval limit: 3.33); it was 1.85 (upper confidence interval limit: 1.56) for 6 h; it was 2.45 (upper confidence interval limit: 2.08) and for ≥ 9 h; indicating that the observed associations were robust against potential unmeasured confounders (Supplementary Fig. 2).

## Discussion

This study is the first to examine the association between sleep duration and healthy aging in a nationally representative sample of older Americans from the BRFSS. After adjusting for potential confounders, an inverted U-shaped relationship was observed: both short and long sleep durations were associated with reduced odds of achieving healthy aging, a pattern consistent across all dimensions of healthy aging. Our findings also revealed interactions between sleep duration and employment status and smoking status on healthy aging. Specifically, the unemployed and current/former smokers with short sleep duration had the lowest odds of achieving healthy aging.

Our study corroborates previous findings on the non-linear relationship between sleep duration and healthy aging [43, 44]. Similar associations were also observed across four dimensions of healthy aging: sleep duration is also nonlinearly associated with major chronic diseases [11], physical function [19, 28], mental health [13], and cognitive function [15]. However, inconsistent relationships between sleep duration and healthy aging have also been observed in other studies. A longitudinal study [45] based on a 19-year follow-up of more than 800 middle-aged and older Americans found that those who slept 7–8 h were 10% less likely to achieve healthy aging than those who slept < 7 or > 8 h at night, but this did not reach statistical significance. Our previous nested case-control study based on the Nurses’ Health Study (NHS) found that, compared to middle-aged and older women who sleep 7 h per day, those who sleep 8 h or more than 9 h per day are less likely to experience healthy aging; similarly, women who sleep 5–6 h or less per day also showed a reduced likelihood of healthy aging, though this association was not statistically significant [19]. A cross-sectional study using 5,616 older adults from the China Health and Retirement Longitudinal Study (CHARLS) [43] found that the relation between sleep duration per night and two dimensions (physiological and cognitive function) was U-shaped, but the relation with disease and disability was statistically significant only in the short sleep group. In our study, it was comprehensively observed that sleep duration had a nonlinear relationship with healthy aging and all dimensions. This inconsistency may arise from differences in study design, sample size, geographic region, representativeness of study populations, sleep duration (nighttime vs. total sleep), and methods used to assess healthy aging. Despite this, most studies suggest that both excessive and insufficient sleep are detrimental to healthy aging [19, 43, 44]. This study found that 7–8 h of sleep may benefit the overall health of older adults, consistent with the National Sleep Foundation’s (NSF)recommendations for this age group [46].

Both insufficient and excessive sleep may reduce the likelihood of healthy aging in older adults through the following mechanisms. First, short sleep duration may induce insulin resistance [47, 48] and reduce leptin secretion [49], thereby increasing calorie intake and impairing blood sugar control. Second, it may also enhance sympathetic nerve activity and disrupt circadian rhythms [50], leading to endocrine and metabolic changes that elevate the risk of obesity and chronic diseases such as diabetes and cancer. Third, sleep deprivation can lead to the deposition of beta-amyloid protein in the brain, which is closely linked to cognitive function [51]. Fourth, individuals with insufficient sleep exhibit elevated inflammation levels [14, 49] and dysregulation of neurotransmitters such as dopamine and serotonin [52], both of which negatively impact mental health. Fifth, those who do not get enough sleep tend to feel fatigued, which in turn reduces physical activity, thus increasing the risk of disability [53].

Studies have shown that sleep duration in the older adults typically decreases naturally with age [54]. Therefore, excessive sleep time contradicts the patterns of normal aging. First, prolonged sleep may lead to abnormal increases in inflammatory markers such as C-reactive protein and interleukin-6 [50], which are closely associated with cardiovascular disease and cancer—both leading causes of mortality. Second, individuals who sleep excessively may often have undiagnosed medical conditions. Third, extended sleep duration can contribute to depression through dysregulation of the hypothalamic-pituitary-adrenal (HPA) axis [14]. Third, long sleep duration is a sign of early neurodegeneration, which may lead to Alzheimer’s disease and dementia. Fourth, those who sleep excessively tend to exercise less, and lack of physical activity is linked to decreased muscle strength and quality in the older persons [55], potentially impairing physiological function [56]. Finally, Insufficient or excessive sleep, both indicators of circadian rhythm disturbances, may alter post-translational protein modifications, such as hyperphosphorylation and acetylation. These changes can impact protein levels [57], which are crucial for maintaining muscle mass and immune function in older adults [58].

Interestingly, this study also found that among the unemployed or current and former smokers, those who slept ≤ 5 h had the lowest odds of achieving healthy aging. One possible explanation is that the unemployed (including retirees) may experience more sleep problems. Additionally, individuals with insufficient sleep are more likely to retire due to health-related issues [59]. Both short sleep duration and tobacco use are components of an unhealthy lifestyle, with inflammation as an overlapping mechanism leading to adverse health outcomes. Current or former smokers are more susceptible to the negative effects of short sleep duration [39]. However, there is currently limited research on the multiplicative interactions of sleep duration with smoking and employment status on healthy aging, and future research is necessary to explore deeper potential mechanisms.

Health professionals should provide tailored sleep hygiene education for the older people, emphasizing regular sleep schedules, and optimizing the sleep environment [60]. Personalized assessments are also advised to identify and mitigate factors affecting sleep, such as psychological stress or chronic conditions [11, 12, 14]. Establishing a stable circadian rhythm and adjusting lifestyle habits may enhance sleep quality and overall health in the older adults.

The study utilized a large, representative sample of older adults in the U.S., further confirmed the U-shaped relationship between sleep duration and healthy aging. However, there are some limitations to this study. First, sleep duration were self-reported and may be subject to recall and social desirability bias [28], but this self-reported sleep duration showed a strong correlation with sleep diary records [61]. Second, the BRFSS telephone survey may have underestimated the prevalence of chronic illnesses [62] and physical limitations [28], as multiple chronic illnesses remain undiagnosed for long periods of time, and the survey excluded homeless populations and those who were unable to answer the telephone due to physical limitations. However, studies have shown no difference in reporting of mental health information between face-to-face and telephone interviews [21]. Telephone interviews may have reduced participants’ embarrassment when discussing mental health. Third, as with any observational study, although we adjusted for major confounders in our analyses, there may still be unknown or residual confounders, such as substance use and pain level. Nevertheless, stratified and sensitivity analyses yielded consistent results, suggesting minimal impact from additional confounders. The calculation of the E value further indicated that unmeasured confounders likely had a negligible effect on the observed associations. Fourth, the cross-sectional design of the BRFSS limits the ability to establish causality. Future large-scale cohort studies are needed to confirm the relationship between sleep duration and healthy aging. Sixth, this study conducted an exploratory (post hoc) stratified analysis with limited sample sizes in some groups; thus, the results should be interpreted with caution. Finally, the study’s findings are based on a U.S. population, limiting generalizability to underdeveloped countries or regions due to cultural and lifestyle differences. However, similar associations between sleep duration and healthy aging have been observed in other countries [43, 63]. Future research should explore interactions between sleep duration and lifestyle factors (e.g., alcohol consumption, diet, physical activity) across diverse cultural contexts.

## Conclusions

In conclusion, in this nationally representative cross-sectional study, an inverted U-shaped relationship was found between sleep duration and healthy aging in the older people. Both short and long sleep duration were associated with lower odds of healthy aging, particularly among the unemployed and smokers, which further complements our previous findings among the American female nurses. To promote healthy aging, this study suggests that older people should sleep 7–8 h per day, aligning with the NSF’s recommendations for older adults. This finding could guide policymakers in developing strategies to enhance sleep health and overall well-being among older adults. In community health management, medical professionals should emphasize sleep duration as a modifiable factor, raising awareness of its importance and incorporating it into long-term healthy lifestyle practices. Additionally, future longitudinal studies across the U.S. are needed to further explore the relationship between sleep duration and healthy aging and potential mediating mechanisms.

## Supplementary Information

Supplementary Material 1.

Supplementary Material 2.

## Data Availability

No datasets were generated or analysed during the current study.

## Abbreviations

ACS: American Community Survey
AI/AN: American Indian/Alaska Native
APA: American Psychiatric Association
BMI: Body Mass Index
BRFSS: Behavioral Risk Factor Surveillance System
CDC: Centers for Disease Control and Prevention
CHARLS: China Health and Retirement Longitudinal Study
CI: Confidence Intervals
DC: District of Columbia
GED: General Educational Development
HPA: Hypothalamic-pituitary-adrenal
ICF: International Classification of Functioning, Disability and Health
NAM: National Academy of Medicine
NHS: Nurses’ Health Study
NSF: National Sleep Foundation
OR: Odds Ratio
PSQI: Pittsburgh Sleep Quality Index
STROBE: Strengthening the Reporting of Observational Studies in Epidemiology
UNGA: United Nations General Assembly
US: United States
