# Supplementary material for: Association between sleep duration and healthy aging among older adults: evidence from the Behavioral Risk Factor Surveillance System
Source: BMC Geriatr. 2026 Feb 21;26:425. doi: 10.1186/s12877-026-07181-8 (PMC13032231; doi:10.1186/s12877-026-07181-8)
Supplement: Supplementary file 2 — Supplementary Material 2. [file 12877_2026_7181_MOESM2_ESM.docx]

**Supplementary Material**

**Association between sleep duration and healthy aging** **among older adults: evidence from the Behavioral Risk Factor Surveillance System**

**Supplementary table 1.** Number and proportion of missing characteristics of the study population

**Supplementary table 2.** Association of sleep duration with four dimensions of healthy aging

**Supplementary table 3.** Association of sleep duration with healthy aging under Model II-based additional adjusted annual household income ($)

**Supplementary table 4.** Association of sleep duration with healthy aging after imputation

**Supplementary fig.1** Spline curve for the association of sleep duration with the 4 dimensions of healthy aging

**Supplementary fig. 2** Curves of the sensitivity analysis for unobserved confounders with E-value highlighted

**Supplemental table 1.** **Number and proportion of missing characteristics of the study population**

| **Characteristics** | **Missing (n, %)** |
| --- | --- |
| Sex | 1, 0.00% |
| Race/ethnicity | 999, 2.85% |
| Marital status | 153, 0.44% |
| Educational attainment | 142, 0.41% |
| Employment status | 140, 0.40% |
| Smoking status | 223, 0.64% |
| Leisure-time physical activity | 64, 0.18% |
| BMI (kg/m^2^) | 1768, 5.04% |

Abbreviations: BMI, body mass index (calculated as weight in kilograms divided by the square of height in meters).

Supplemental table 2. Association of sleep duration with four dimensions of healthy aging

| **Outcome,**  sleep duration, h | **No.** | **OR(95%CI), *P*** | | | |
| --- | --- | --- | --- | --- | --- |
|  |  | **Healthy aging,**  **No. (%)** | **Crude model, (n=35,056)** | **Adjusted model I (*n*=33,659)** | **Adjusted model Ⅱ (*n*=30,648)** |
| **No major chronic diseases** | | | | | |
| ≤5 | 2,701 | 371 (13.7) | **0.47 (0.42,0.53), <0.05** | **0.53(0.47,0.60), <0.001** | **0.59 (0.52, 0.68), <0.001** |
| 6 | 6,026 | 1216 (20.2) | **0.74 (0.69,0.80), <0.001** | **0.78(0.72,0.85), <0.001** | **0.83 (0.76, 0.90), <0.001** |
| 7 | 9,958 | 2524 (25.4) | 1.00 | 1.00 | 1.00 |
| 8 | 12,118 | 3052 (25.2) | 0.99 (0.93,1.05), 0.784 | 1.04(0.97,1.10), 0.843 | 1.06 (0.99, 1.13), 0.098 |
| ≥9 | 4,253 | 775 (18.2) | **0.66 (0.60,0.72), <0.001** | **0.73(0.66,0.79), <0.001** | **0.76 (0.69, 0.84), <0.001** |
| **No physical impairment** | | | | | |
| ≤5 | 2,701 | 1057 (39.1) | **0.30 (0.28,0.33), <0.001** | **0.36(0.33,0.40), <0.001** | **0.42 (0.38, 0.46), <0.001** |
| 6 | 6,026 | 3429 (56.9) | **0.62 (0.58,0.67), <0.001** | **0.66(0.62,0.71), <0.001** | **0.73 (0.68, 0.79), <0.001** |
| 7 | 9,958 | 6764 (67.9) | 1.00 | 1.00 | 1.00 |
| 8 | 12,118 | 7637 (63.0) | **0.80 (0.76,0.85), <0.001** | **0.84(0.79,0.89), <0.001** | **0.87 (0.82, 0.92), <0.001** |
| ≥9 | 4,253 | 2004 (47.1) | **0.42 (0.39,0.45), <0.001** | **0.47(0.44,0.51), <0.001** | **0.53 (0.49, 0.58), <0.001** |
| **No subjective cognitive impairment** | | | | | |
| ≤5 | 2,701 | 2252 (83.4) | **0.47 (0.41,0.53), <0.001** | **0.55(0.48,0.62), <0.001** | **0.56 (0.49, 0.65), <0.001** |
| 6 | 6,026 | 5376 (89.2) | **0.77 (0.69,0.86), <0.001** | **0.82(0.73,0.91), <0.001** | **0.87 (0.78, 0.98), 0.024** |
| 7 | 9,958 | 9110 (91.5) | 1.00 | 1.00 | 1.00 |
| 8 | 12,118 | 11007 (90.8) | 0.92 (0.84,1.01), 0.090 | 0.97(0.88,1.07), 0.619 | 0.99 (0.89, 1.09), 0.834 |
| ≥9 | 4,253 | 3585 (84.3) | **0.50 (0.45,0.56), <0.001** | **0.56(0.50,0.63), <0.001** | **0.60 (0.53, 0.68), <0.001** |
| **No depression** | | | | | |
| ≤5 | 2,701 | 1826 (67.6) | **0.38 (0.35,0.42), <0.001** | **0.45(0.41,0.50), <0.001** | **0.49 (0.44, 0.55), <0.001** |
| 6 | 6,026 | 4783 (79.4) | **0.70 (0.65,0.76), <0.001** | **0.76(0.70,0.83), <0.001** | **0.82 (0.74, 0.89), <0.001** |
| 7 | 9,958 | 8419 (84.6) | 1.00 | 1.00 | 1.00 |
| 8 | 12,118 | 10138 (83.7) | 0.94 (0.87,1.01), 0.074 | 0.98(0.91,1.05), 0.179 | 0.98 (0.90, 1.06), 0.597 |
| ≥9 | 4,253 | 3086 (72.3) | **0.48 (0.44,0.53), <0.001** | **0.53(0.49,0.58), <0.001** | **0.56 (0.51, 0.62), <0.001** |

Abbreviations: AI/AN: American Indian/Alaska Native; BMI, body mass index (calculated as weight in kilograms divided by the square of height in meters); OR, odds ratio; CI, conﬁdence interval.

Model I was adjusted for age group (65-69, 70-74, ≥75 years), sex (men, women), race/ethnicity (White, Black, Hispanic, American Indian/Alaska Native (AI/AN), Asian, Other race/Multiracial), marital status (married/living with partner, previously married, never married), educational attainment (<high school graduate, high school graduate/GED, some college/technical school, college graduate) and employment status (employed, unemployed, unable to work, student/homemaker/retired).

Model II was additionally adjusted for smoking status (current smoker, former smoker, never smoker), leisure-time physical activity (yes, no), BMI (<18.5 kg/m^2^, 18.5-24.9 kg/m^2^, 25.0-25.9 kg/m^2^, ≥30.0 kg/m^2^).

**Supplemental table 3.** **Association of sleep duration with healthy aging under Model II-based additional adjusted annual household income ($) ^a^**

| Sleep duration, hours | No. | Healthy aging, No. (%) | *OR*(95%*CI*) | | |
| --- | --- | --- | --- | --- | --- |
|  |  |  | Crude model, (*n*=35,056) | Adjusted model I, (*n=*33,659) | Adjusted model Ⅱ, (*n*=25,134) |
| ≤5 | 2,701 | 224(8.3) | **0.37 (0.32,0.43), <0.001** | **0.51 (0.43, 0.59), <0.001** | **0.52 (0.44, 0.62), <0.001** |
| 6 | 6,026 | 878(14.6) | **0.70 (0.64,0.77), <0.001** | **0.79 (0.72, 0.87), <0.001** | **0.82 (0.74, 0.91), <0.001** |
| 7 | 9,958 | 1948(19.6) | 1.00 | 1.00 | 1.00 |
| 8 | 12,118 | 2250(18.6) | 0.94 (0.88,1.00), 0.060 | 0.96 (0.90, 1.03), 0.480 | 0.99 (0.92, 1.08) |
| ≥9 | 4,253 | 482(11.3) | **0.53 (0.47,0.58), <0.001** | **0.65 (0.58, 0.73), <0.001** | **0.65 (0.57, 0.74), <0.001** |

**^a^** Annual household income (<35,000**$**/ 35,000–75,000**$**/ >75,000**$**)
Abbreviations: AI/AN: American Indian/Alaska Native; BMI, body mass index (calculated as weight in kilograms divided by the square of height in meters); OR, odds ratio; CI, conﬁdence interval.

Model I was adjusted for age group (65-69, 70-74, ≥75 years), sex (men, women), race/ethnicity (White, Black, Hispanic, AI/AN, Asian, other race/multiracial), marital status (married/living with partner, previously married, never married), educational attainment (<high school graduate, high school graduate/GED, some college/technical school, college graduate) and employment status (employed, unemployed, unable to work, student/homemaker/retired).

Model II was additionally adjusted for smoking status (current smoker, former smoker, never smoker), leisure-time physical activity (yes, no), BMI (<18.5 kg/m^2^, 18.5-24.9 kg/m^2^, 25.0-25.9 kg/m^2^, ≥30.0 kg/m^2^).

**Supplemental table 4.** **Association of sleep duration with healthy aging after imputation**

| Sleep duration, hours | No. | Healthy aging, No. (%) | *OR*(95%*CI*) | | |
| --- | --- | --- | --- | --- | --- |
|  |  |  | Crude model, (*n*=35,056) | Adjusted model I, (*n*=35,056) | Adjusted model Ⅱ (*n*=35,056) |
| ≤5 | 2,701 | 224(8.3) | **0.37 (0.32,0.43), <0.001** | **0.42 (0.37, 0.49), <0.001** | **0.49 (0.37, 0.49), <0.001** |
| 6 | 6,026 | 878(14.6) | **0.70 (0.64,0.77), <0.001** | **0.72 (0.66, 0.78), <0.001** | **0.78 (0.71, 0.85), <0.001** |
| 7 | 9,958 | 1948(19.6) | 1.00 | 1.00 | 1.00 |
| 8 | 12,118 | 2250(18.6) | 0.94 (0.88,1.00), 0.061 | 0.99 (0.92, 1.06), 0.669 | 1.02 (0.95, 1.09), 0.595 |
| ≥9 | 4,253 | 482(11.3) | **0.53 (0.47,0.58), <0.001** | **0.58 (0.52, 0.65), <0.001** | **0.65 (0.58, 0.73), <0.001** |

Abbreviations: AI/AN: American Indian/Alaska Native; BMI, body mass index (calculated as weight in kilograms divided by the square of height in meters); OR, odds ratio; CI, conﬁdence interval.

Model I was adjusted for age group (65-69, 70-74, ≥75 years), sex (men, women), race/ethnicity (White, Black, Hispanic, American Indian/Alaska Native (AI/AN), Asian, Other race/Multiracial), marital status (married/living with partner, previously married, never married), educational attainment (<high school graduate, high school graduate/GED, some college/technical school, college graduate) and employment status (employed, unemployed, unable to work, student/homemaker/retired).

Model II was additionally adjusted for smoking status (current smoker, former smoker, never smoker), leisure-time physical activity (yes, no), BMI (<18.5 kg/m^2^, 18.5-24.9 kg/m^2^, 25.0-25.9 kg/m^2^, ≥30.0 kg/m^2^).


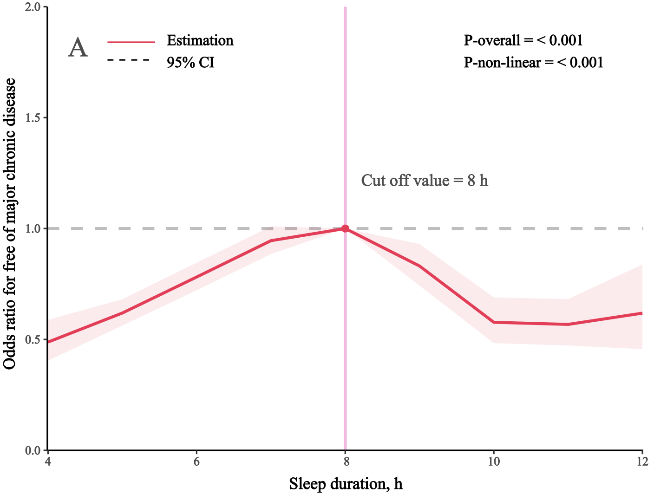

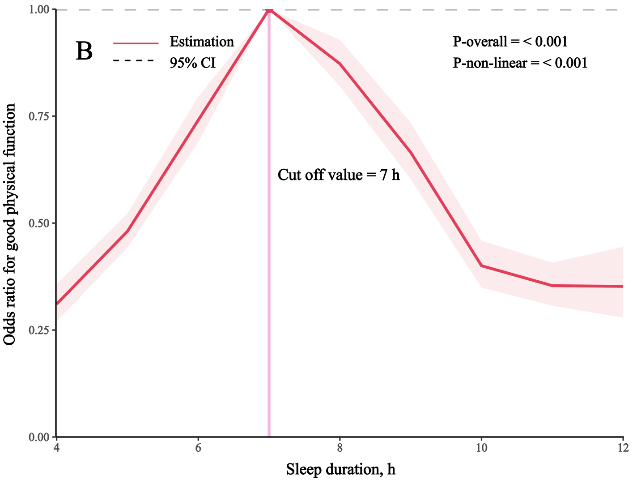


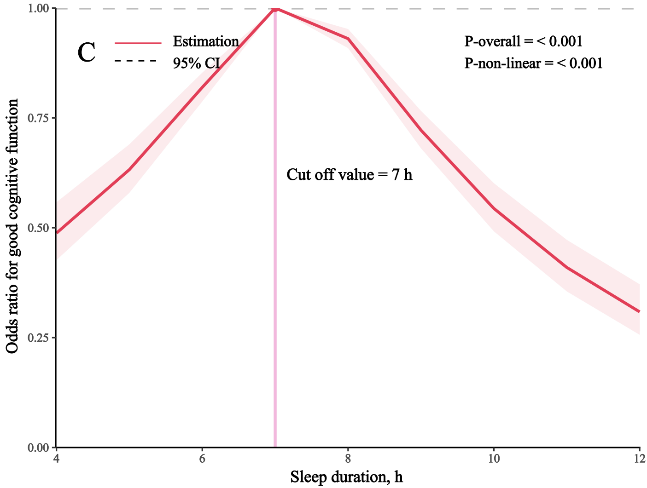

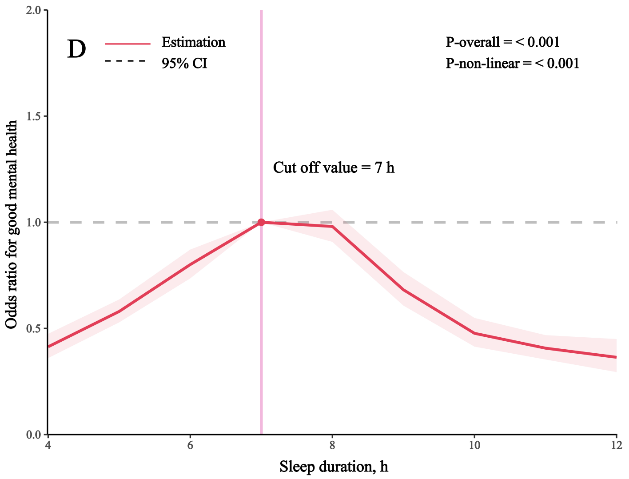


**Supplemental fig.** **1 Spline curve for the association of sleep duration with the 4 dimensions of healthy aging.** (A) sleep duration and free of chronic diseases, (B) sleep duration and good physical function, (C) sleep duration and good mental health, (D) sleep duration and good cognitive function.

Adjusted for age group (65-69, 70-74, ≥75 years), sex (men, women), race/ethnicity (White, Black, Hispanic, American Indian/Alaska Native (AI/AN), Asian, Other race/Multiracial), marital status (married/living with partner, previously married, never married), educational attainment (<high school graduate, high school graduate/GED, some college/technical school, college graduate), employment status (employed, unemployed, unable to work, student/homemaker/retired), smoking status (current smoker, former smoker, never smoker), leisure-time physical activity (yes, no), BMI (<18.5 kg/m^2^, 18.5-24.9 kg/m^2^, 25.0-25.9 kg/m^2^, ≥30.0 kg/m^2^).

**
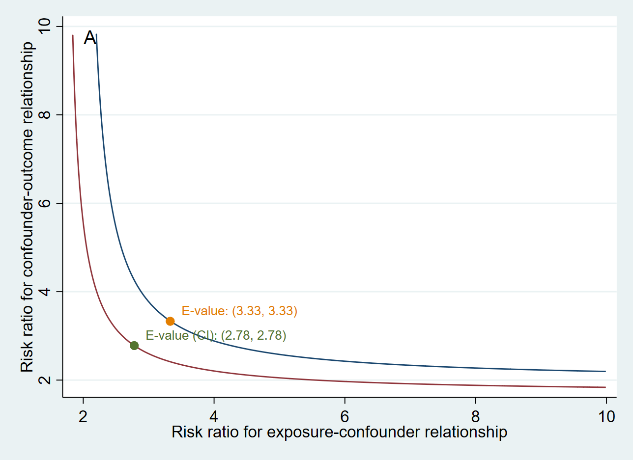

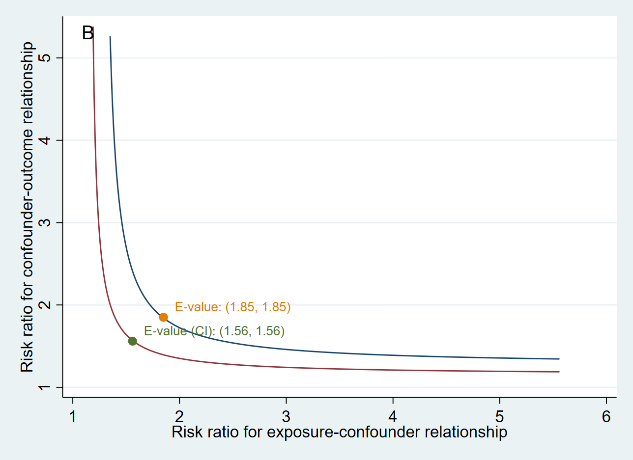
**

**
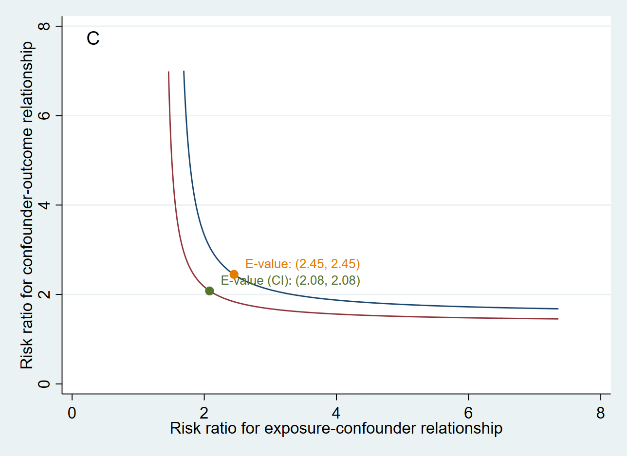
**

**Supplemental fig. 2** **Curves of the sensitivity analysis for unobserved confounders with *E*-value highlighted (A**: **≤5h *VS* 7h; B: 6h *VS* 7h; C: ≥9h *VS* 7h)**.
